# Supplementary material for: The photolyase/cryptochrome of Aspergillus nidulans senses oxidative stress and shuttles from nuclei to mitochondria
Source: Nat Commun. 2026 Feb 7;17:1483. doi: 10.1038/s41467-026-69403-2 (PMC12887071; doi:10.1038/s41467-026-69403-2)
Supplement: Supplementary file 1 — Supplementary Information [file 41467_2026_69403_MOESM1_ESM.pdf]

Supplementary information

The photolyase/cryptochrome of *Aspergillus nidulans* senses oxidative stress and shuttles from nuclei to mitochondria

Alexander Landmark, Tim Rudolf, Kevin Hundshammer, Jasmin Böhm, Kai Leister, Sylvia Erhardt and Reinhard Fischer

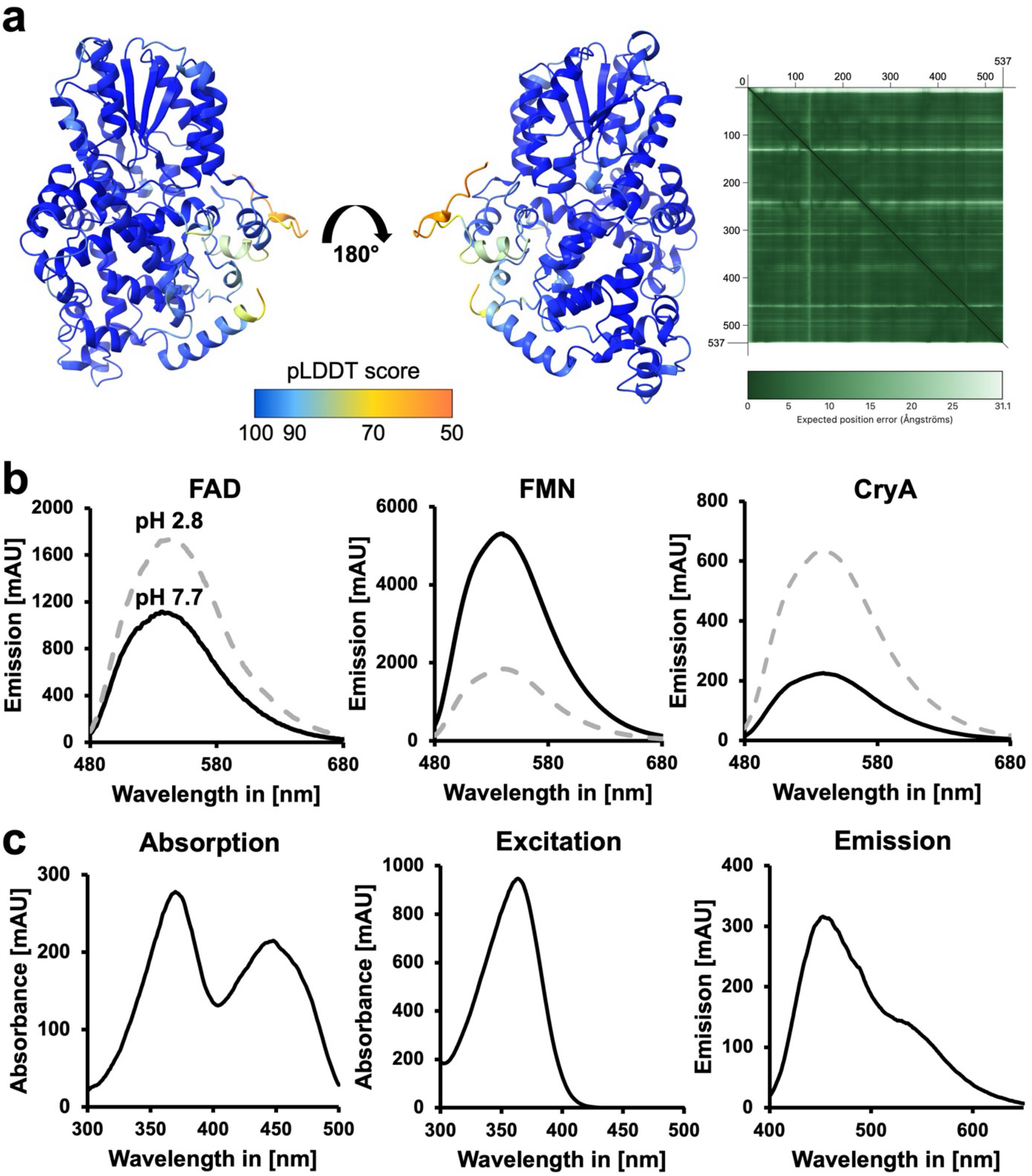

Supplementary Fig. 1: Structural and spectroscopic analysis of CryA. (a) Structural AlphaFold prediction of CryA with overlaid pLDDT scores from two viewing angles. Respective PAE map is shown on the right **(b)** Emission spectrum of FAD and FMN standards and the isolated chromophore of CryA. FAD and FMN were used with a concentration of 10 µM. The chromophore of CryA was separated from the protein by boiling the sample for 5 min and then centrifugation for 15 min at 4°C. All samples were in 0.1 M potassium phosphate buffer with a pH of 7.7, which was also used as baseline. The spectrum was measured before and after the addition of 100 µl 1 M HCl to acidify the solution to pH 2.8. The excitation wavelength was 450 nm and recorded between 480 nm to 680 nm with a high sensitivity, a scan speed of 200 nm/min and an excitation and emission bandwidth of 2.5 nm. All samples were kept in the dark and experiments were performed under red light on the spectrofluorometer. **(c)** Absorption, excitation, and emission spectrum of isolated chromophore from CryA. The chromophores of CryA were separated from the protein by boiling the sample

for 5 min and then centrifugation for 15 min at 4°C. All samples were in 0.1 M potassium phosphate buffer with a pH of 7.7, which was also used as baseline. The absorption spectrum was measured on the spectrophotometer. The emission wavelength of the excitation spectrum was 450 nm and the excitation wavelength for the emission spectrum was 365 nm. All spectra were recorded with high sensitivity, a scan speed of 200 nm/min and an excitation and emission bandwidth of 2.5 nm. All samples were kept in the dark, and experiments were performed under red light. Source data are provided as a Source Data file.

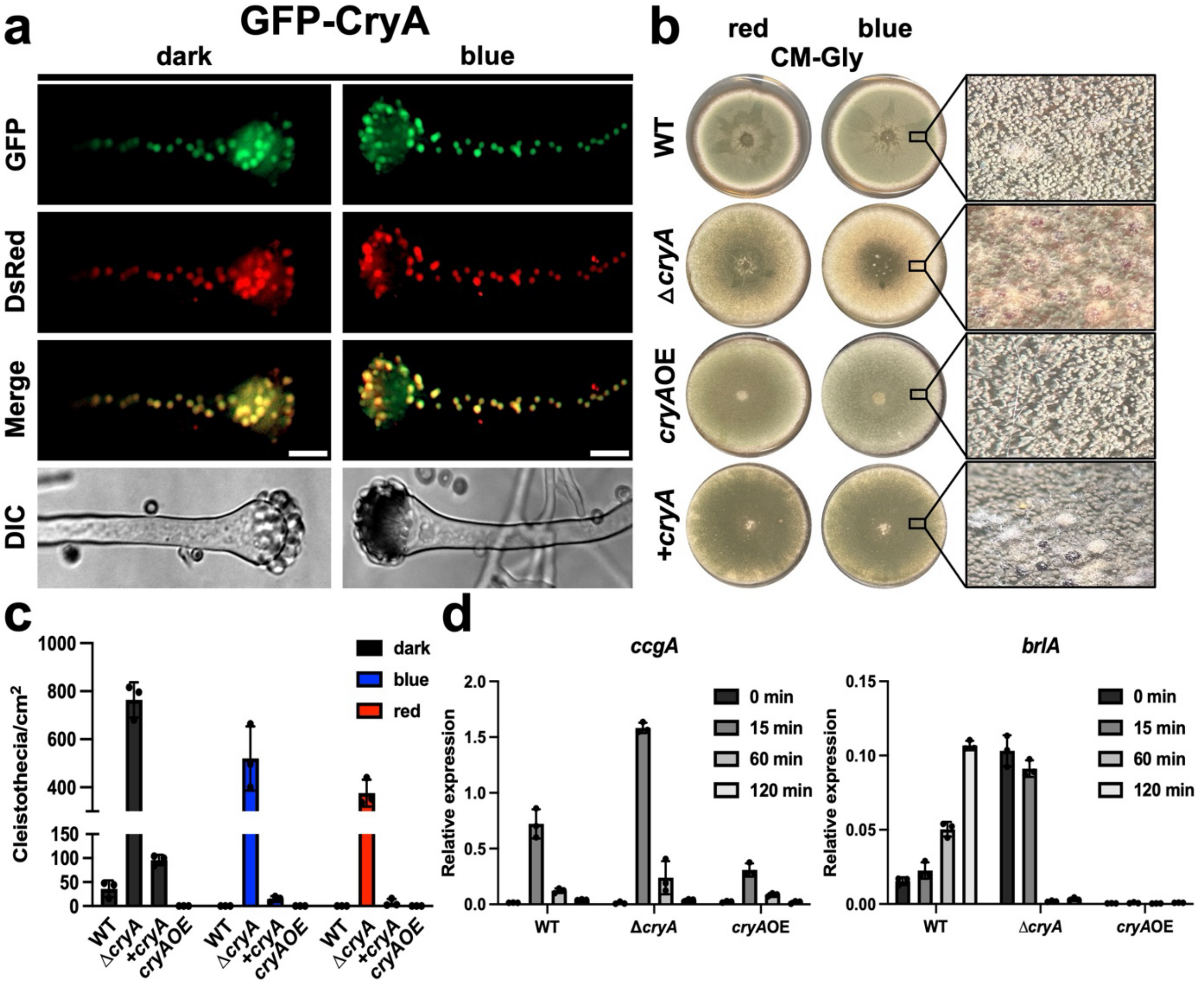

Supplementary Fig. 2: Sexual and asexual development of *cryA* mutants. (a) Localization of CryA in conidia with different light conditions. CryA was fused N-terminally to GFP under the control of the *alcA* promoter. Spores were incubated overnight at 37°C in solid minimal media with 2% threonine instead of glucose to induce the promoter. Nuclei were visualized with the NLS of *stuA* fused to DsRed. The scale bar represents 10 µm. **(b)** Growth assay of wild-type, the Δ*cryA* strain, the *cryA*OE strain, and the re-complementation strain +*cryA* on CM-media. 10^5 spores were inoculated on complete media with 1% glycerol (CM-Gly) and grown for 7 days at 37°C illuminated with red (left) or blue (right) light (200 µmol photons/m^2*s^2). Microscopy was performed on a Stemi DV4. **(c)** Cleistothecia count of the *cryA* mutants under different light conditions. For each strain and condition, biological triplicates and technical quadruplicates were used. Cleistothecia were counted in a 3 cm radius around the center of the plate, and the counting spots were randomized. **(d)** Photoadaptation assay of WT, Δ*cryA*, and the *cryA*OE. 1.5*10^8 spores of the respective strains were incubated for 16 h in 10 ml minimal media with 2% threonine at 37°C in the dark. Light-treated samples were illuminated for the indicated minutes with blue- and red light (200 µmol photons/m^2*s^2) simultaneously before harvesting the mycelia under green safety light. For the qRT-PCR *h2b* was used as housekeeping gene for normalization. Error bars represent the standard deviation of three biological and two technical replicates. Source data are provided as a Source Data file.

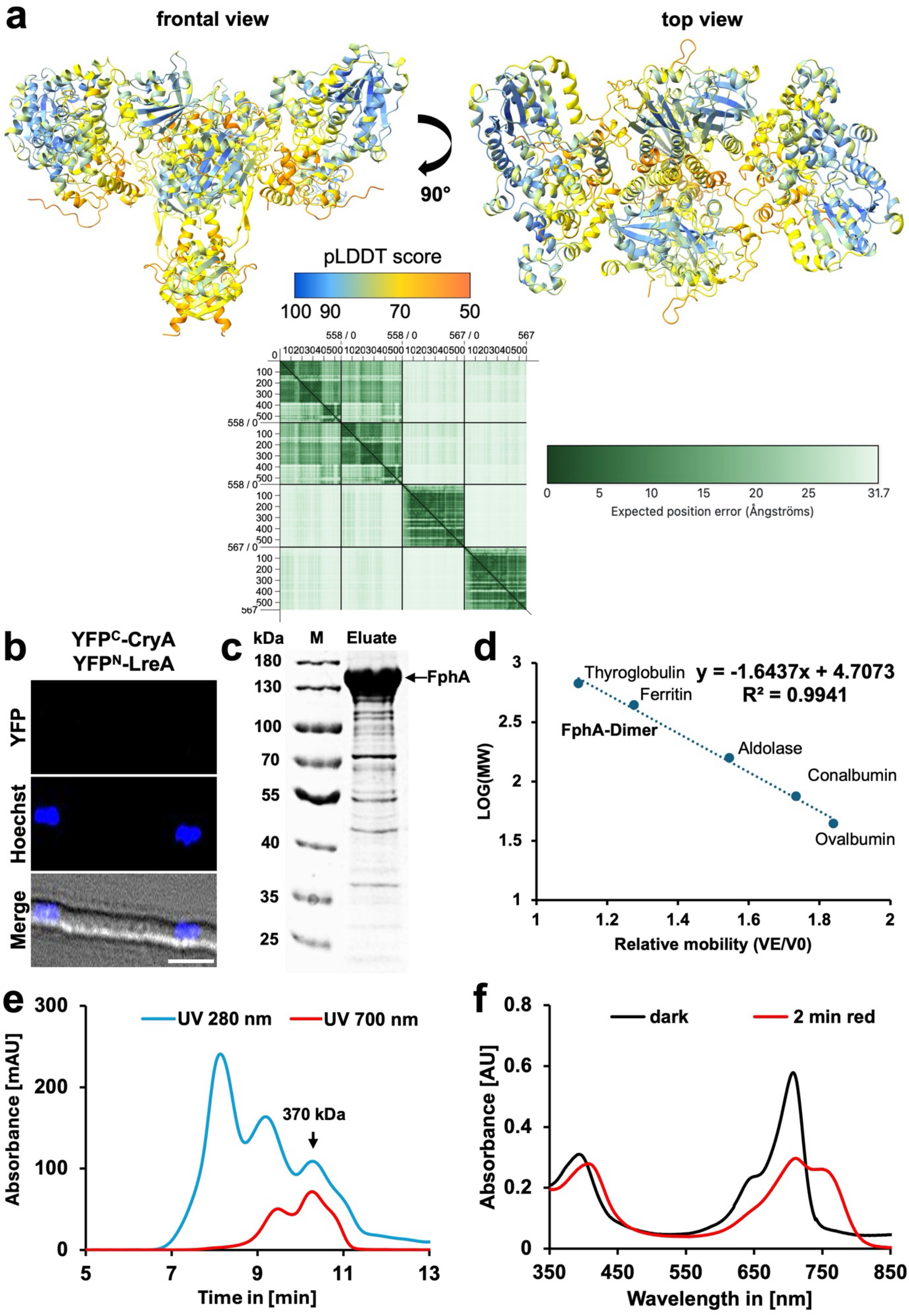

Supplementary Fig. 3: Interaction of FphA with CryA. (a) Structural AlphaFold prediction of CryA together with fphA with overlaid pLDDT scores from two viewing angles.

Respective PAE map is shown below. **(b)** BiFC assay with CryA/LreA as negative control for the CryA/FphA interaction. Strains were incubated overnight at 25°C in minimal media with 2% threonine to induce the promoter. HOECHST staining was used for nuclei visualization. The scale bar represents 5 µm. **(b)** SDS-PAGE of heterologously expressed and purified FphA from *E. coli.* PageRuler 180 kDa was used as protein standard. **(c)** Calibration of the Size exclusion chromatography (SEC) column with known protein standards (blue) was used to identify the purified phytochrome dimer after SEC (red). Standards were Thyroglobulin (669 kDa), Ferritin (440 kDa), Aldolase (158 kDa), Conalbumin (75 kDa), and Ovalbumin (44 kDa). **(d)** SEC of purified FphA. UV light of 280 nm (blue) and 700 nm (red) was used to detect functional FphA as dimer (size with tag: ~284 kDa, calculated size after SEC: ~370 kDa). **(e)** Absorption spectrum of the FphA dimer (concentration of 14 µM) after SEC. The sample was kept in the dark (black line) or illuminated for 2 min with red light (red line) before the measurement. Source data are provided as a Source Data file.

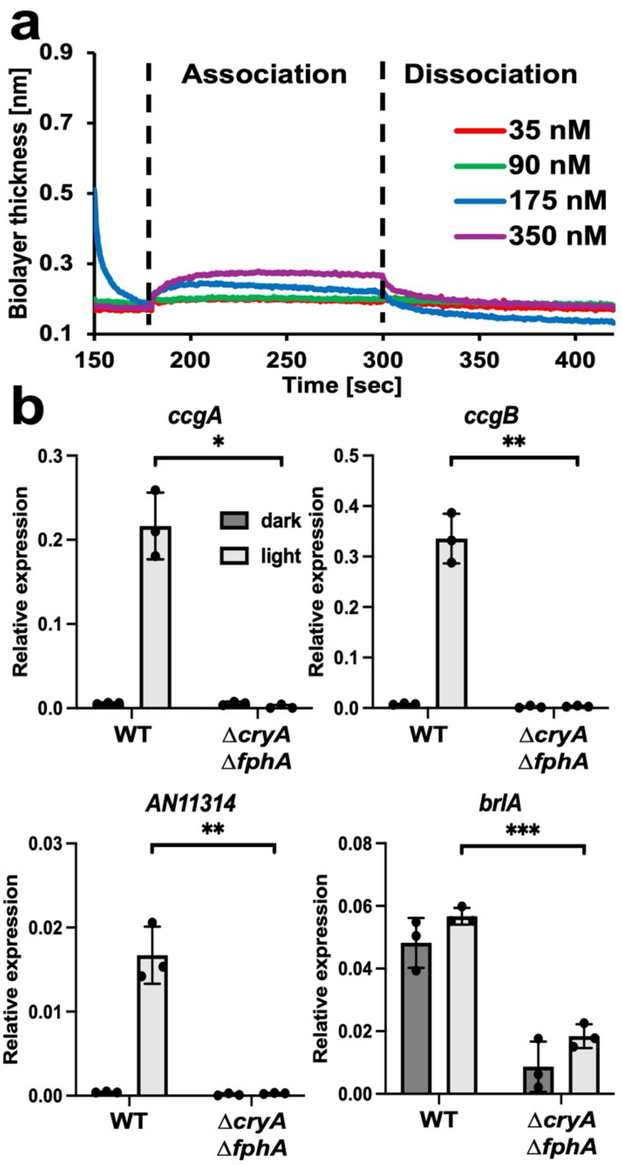

Supplementary Fig. 4: Interaction of CryA and FphA is chromophore dependent (a) Biolayer interferometry analysis with purified CryA with 6xHis-tag and $FphA^{C195A}$ with Strep-tag. CryA was immobilized as bait with a concentration of 30 µg/ml on a Nickel-nitrilotriacetic acid (NTA) biosensor. Phytochrome was prepared at the indicated concentrations, and association/dissociation kinetics were recorded to calculate the K_d value with the BLI software of the system. All protein solutions were prepared in BLI buffer. For the analysis, a global fit with a step correction for the start of the association and dissociation were selected. As reference BLI buffer without protein was used. **(b)** Expression analysis of genes responding to phytochrome in wild type and a Δ*cryA*Δ/*fphA*-double deletion strain. $1.5*10^8$ spores of the respective strains were incubated for 16 h in 10 ml minimal media at 37°C in the dark. Light treated samples were illuminated for 15 minutes with blue- and red light (200 µmol photons/m^{2}*s^{2}) simultaneously before harvesting the mycelia under green safety light. For the qRT-PCR *h2b* was used as housekeeping gene for normalizing the expression levels. Error bars represent the standard deviation of three biological and two technical replicates. For statistical analysis, a two-tailed Student's t-test was performed, * p≤0.05, **p≤0.01, ***p≤0.001. Dots represent each individual biological replicate. Source data are provided as a Source Data file.

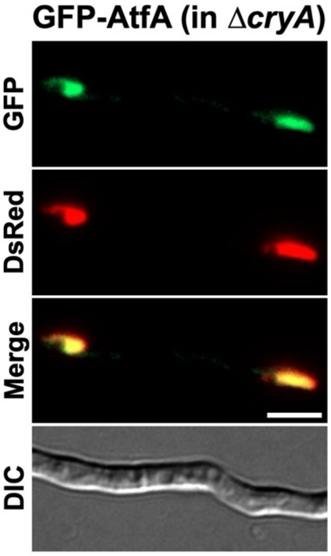

Supplementary Fig. 5: AtfA localizes in the nucleus in the absence of CryA. The strain was incubated overnight at 25°C in minimal media with 2% threonine to induce the promoter. DsRed fused C-terminally to the NLS of StuA was used for nuclei visualization. The scale bar represents 5 µm.

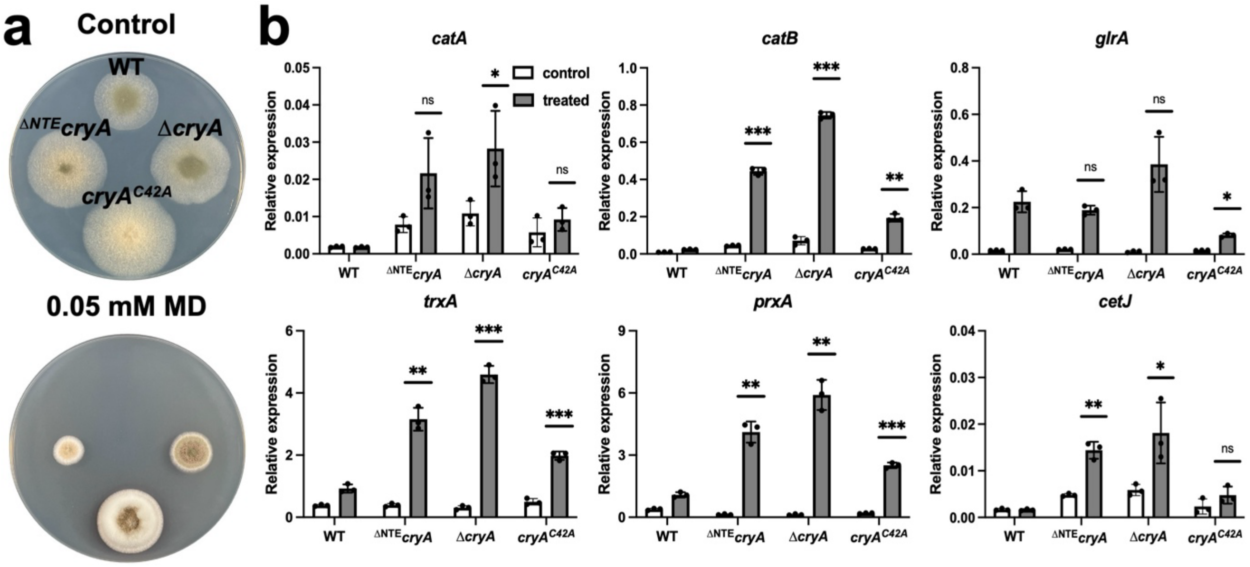

Supplementary Fig. 6: Analysis of the stress response to menadione in the WT, the *cryA* deletion strain, the truncation mutant $^{\Delta NTE}$*cryA*, and the cysteine mutant *cryA*^{C42A}. (a) Comparison of wild-type colonies with the *cryA* deletion, the Δ*cryA* strain re-complemented with an overexpression mutant of the N-terminal truncation mutant of CryA ($^{\Delta NTE}$*cryA*), and re-complementation of the Δ*cryA* strain with the cysteine mutant (*cryA*^{C42A}). 1,000 spores were grown for 5 days at 30°C on minimal media with 2% threonine in light (white light with 200 µmol photons/m^2*s^2) with no supplements (control) or 0.05 mM menadione (MD) **(b)** Expression analysis of genes responding to oxidative stress in wild type, the $^{\Delta NTE}$*cryA* strain, and the *cryA*^{C42A} strain. 1.5*10^8 spores of the respective strains were incubated for 16 h in 10 ml minimal media at 37°C completely in the dark. Before harvesting, the mycelia were transferred to pre-warmed liquid minimal media without supplements (control, white) or 0.05 mM menadione (treated, grey) under green safety light and incubated for 25 minutes. For the qRT-PCR *h2b* was used as housekeeping gene for normalizing the expression level. Error bars represent the standard deviation of three biological and two technical replicates. For statistical analysis, a two-tailed Student's t-test was performed and compared to the wild-type and the respective mutants, ^{ns}p>0.05; * p≤0.05, **p≤0.01, ***p≤0.0001. Dots represent the individual biological replicates. Source data are provided as a Source Data file.

Supplementary Table 1: List of used CPF members with their respective organisms, the correspondent CPD subfamily, and the UniProt ID.

Name	Organism	CPD subfamily	UniProt ID (Last accessed: 31 Oct 2024)
A. alt Phr1	*Alternaria alternata*	CPD photolyases	A0A177DDI4
A. cla CPD Phr1	*Aspergillus clavatus*	CPD photolyases	A1CSD2
A. cla Cry2	*Aspergillus clavatus*	Animal cryptochrome (ACRY)	A1CJL8
A. fab PhrA	*Agrobacterium fabrum*	Plant cryptochrome (PCRY)	A9CJC9
A. fla CPD Pho	*Aspergillus flavus*	CPD photolyases	A0A364LX54
A. fum CPD Pho	*Aspergillus fumigatus*	CPD photolyases	A0A9P8NN75
A. nid CryA	*Aspergillus nidulans*	CPD photolyases	Q5BGE3
A. nig Cry2	*Aspergillus niger*	Animal cryptochrome (ACRY)	A0A9W5ZSF5
A. nig PHR1	*Aspergillus niger*	CPD photolyases	A0A9W5ZU83
A. tha Cry1	*Arabidopsis thaliana*	Plant cryptochrome (PCRY)	Q43125
A. tha Cry2	*Arabidopsis thaliana*	Plant cryptochrome (PCRY)	Q96524
A. tha Cry3	*Arabidopsis thaliana*	CRY-DASH	Q84KJ5
A. tha PHR	*Arabidopsis thaliana*	CPD photolyases	Q9SB00
B. cin Cry1	*Botrytis cinerea*	CPD photolyases	A0A384JIP4
B. cin Cry2	*Botrytis cinerea*	CRY-DASH	A0A384JRT3
C. rei CPH1	*Chlamydomonas reinhardtii*	Plant cryptochrome (PCRY)	Q42696
N. cra CRY	*Neurospora crassa*	CRY-DASH	Q7SI68

N. cry PHR	*Neurospora crassa*	CPD photolyases	A1Z757
D. mel CPD Pho	*Drosophila melanogaster*	CPD photolyases	P00914
D. mel Cry1	*Drosophila melanogaster*	Animal cryptochrome (ACRY)	O77059
E. col PHR	*Escherichia coli*	CPD photolyases	Q16526
H. sap Cry1	*Homo sapiens*	Animal cryptochrome (ACRY)	S2K8G5
M. cir CryA	*Mucor circinelloides*	CRY-DASH	A0A4P7N0U2
M. ory Phr	*Magnaporthae oryzae*	CPD photolyases	P27526
P. bla CRY-DASH	*Phycomyces blakesleeanus*	Plant cryptochrome (PCRY)	A0A0F7W019
S. cer PHR	*Saccharomyces cerevisiae*	CPD photolyases	P05066
S. scl Cry1	*Sclerotinia slerotiorum*	CRY-DASH	A7F2C9
S. scl CRY-DASH	*Sclerotinia slerotiorum*	CRY-DASH	A0A1D9Q9S6
Syn. sp CryD	*Synechocystes sp.*	CRY-DASH	P77967
Syn. sp PHR	*Synechocystes sp.*	CPD photolyases	P05327
T. atr PHR1	*Trichoderma atroviride*	CPD photolyases	G9PA82
T. the PHR	*Thermus thermophilus*	CPD photolyases	P61497
U. may CRY-DASH	*Ustilago maydis*	CRY-DASH	A0A0D1DUB1
X. lae CRY-DASH	*Xenopus laevis*	CRY-DASH	Q75WS4

Supplementary Table 2: *A. nidulans* strains used in this study.

Strain	Genotype	Reference
sKV103	*pyrG89; pyroA4; veA+*	[1]
sJR2	*pyrG89; pyroA4; veA+; ∆nku::argB*	[2]
sJP2	*pyroA4*; *∆fphA::argB, veA+*	[3]
sKL15	*pyroA4; alcA(p)::stuA-NLS::DsRed*	[4]
sAUL1	*alcA(p)::YFPC::cryA*<br>*alcA(p)::YFPN::fphA* in sKV103	This work
sAUL2	*alcA(p)::cryA::trpC(t)* in sKV103	This work
sAUL3	*alcA(p)::GFP::cryA* in sKV103	This work
sAUL6	*alcA(p)::YFPN::cryA*<br>*alcA(p)::YFPC::fphA* in sKV103	This work
sAUL11.2	*∆cryA* in sJR2	This work
sAUL20	*alcA(p)::YFPC::cryA*<br>*alcA(p)::YFPN::atfA* in sKV103	This work
sAUL21	*alcA(p)::YFPN::cryA*<br>*alcA(p)::YFPC::atfA* in sKV103	This work
sAUL23	*∆cryA, ∆fphA*<br>Crossing between sAUL11.2 and sJP2	This work
sAUL24	*alcA(p)::GFP::∆NTE20cryA* in sKV103	This work
sAUL25	*alcA(p)::GFP::∆NTE40cryA* in sKV103	This work
sAUL26	*alcA(p)::GFP::∆NTE60cryA* in sKV103	This work
sAUL30	*alcA(p)::∆NTE70cryA::trpC(t)* in sAUL11.2	This work
sAUL32	*alcA(p)::GFP::*$cryA^{C42A}$ in sKV103	This work
sAUL33	*cryA(p)::cryA* in sAUL11.2	This work
sAUL34	*alcA(p)::YFPC::cryA*<br>*alcA(p)::YFPN::fphA* in sKL15	This work

sAUL35	*alcA(p)::YFPN::cryA*<br>*alcA(p)::YFPC::fphA* in sKL15	This work
sAUL36	*alcA(p)::GFP::atfA* in sAUL11.2	This work
sAUL37	*alcA(p)::cryA^{C42A}::trpC(t)* in sAUL11.2	This work
sAUL38	*alcA(p)::GFP::cryA* in sKL15	This work

Supplementary Table 3: Plasmids used in this study.

Name	Description	Reference
pJP4	*alcA(p)::YFPN::fphA, pyro*	[3]
pJP5	*alcA(p)::YFPC::fphA, pyrG*	[3]
pAA15	*tet(p)::fphAsyn::strep-tag,* Ampicillin	[5]
pAUL3	*alcA(p)::YFPC::cryA, pyrG*	This work
pAUL4	*alcA(p)::YFPN::cryA, pyro*	This work
pAUL5	*alcA(p)::cryA::trpC(t), pyrG*	This work
pAUL6	*alcA(p)::GFP::cryA, pyrG*	This work
pAUL12	*ΔcryA x pJEt1.2, pyrG*	This work
pAUL13	*T7(p)::6xHis-tag::cryA::T7(t),* Kanamycin	This work
pAUL15	*cryA(p)::cryA*	This work
pAUL32	*alcA(p)::GFP::Δ20NTE-cryA*	This work
pAUL33	*alcA(p)::GFP::Δ40NTE-cryA*	This work
pAUL34	*alcA(p)::GFP::Δ60NTE-cryA*	This work
pAUL44	*alcA(p)::Δ70NTE-cryA::trpC(t)*	This work
pAUL93	*alcA(p)::*$cryA^{C42A}$*::trpC(t)*	This work
pALBL33	*alcA(p)::YFPN::atfA*	This work
pALBL34	*alcA(p)::YFPC::atfA*	This work

pAUL103	*tet(p)::fphAsyn*^{C195A}*::strep-tag,* Ampicillin	This work
pAUL104	*alcA(p)::GFP::atfA*	This work

Supplementary Table 4: Oligonucleotides used in this study.

Name	Sequence 5´ to 3´	Description
CryA YFPC Efi for	catgaaccacGGCGCGCCgATGCGGCAGAAGCGCAAAC	N-terminal YFPC fusion
CryA YFPN Efi for	CCATCGCCACGGCGCGCCGATGCGGC AGAAGCGCAAAC	N-terminal YFPN fusion
CryA GFP Efi for	GAACTATACAAAGGCGCGCCGATGCGGCAGAAGCGCAAAC	N-terminal GFP fusion
CryA GFP/YFP Efi rev	ctagaggatccTTAATTAATTATGCTCCCCGCGCAGC	N-terminal YFP/GFP fusion
CryA -GFP +trpC Efi for	CAGTTAATTAGGGCGCGCCgATGCGGCAGAAGCGCAAAC	Overexpression backbone
CryA -GFP +trpC Efi rev	gtaacgttaagtTTAATTAATTATGCTCCCCGCGCAGC	Overexpression backbone
CryA pET28a for	ggtgccgcgcggcagccatATGCGGCAGAAGCGCAAAC	Heterologous expression of CryA
CryA pET28a rev	gtggtggtggtggtgctcgaGTGCTCCCCGCGCAGCATCC	Heterologous expression of CryA
pyrG ΔcryA 2000 bp US for	AAACTTCGCAAGCTTGGAACGATCTTCATCATTCGTCGCTTTC	Homologous recombination of CryA
pyrG ΔcryA 2000 bp DS rev	TATGAACTGGACCTGTATATACTGCTTCCTCAACCTCTCGA	Homologous recombination of CryA
ΔcryA 2000 bp US for	GGCTCGAGTTTTTCAGCAAGATCTAGAACCATGTACGTGCTAAAAC	Homologous recombination of CryA
ΔcryA 2000 bp US rev	GTTCCAAGCTTGCGAAGTTTATTTG	Homologous recombination of CryA
ΔcryA 2000 bp DS for	TATATACAGGTCCAGTTCATATAACG	Homologous recombination of CryA
ΔcryA 2000 bp DS rev	AGGAGATCTTCTAGAAAGATGAAGGCCGCATGCCACCATCGCAT	Homologous recombination of CryA
ΔcryA PyrG Forward Primer Kontrolle	GAGCTCTCGTCAAGAGTGGAA	Test primer for Δ*cryA*
ΔcryA PyrG Reverse Primer Kontrolle	CATTTTGGGCCAAGGGTTTCTC	Test primer for Δ*cryA*

CryA N-trunc 20 GFP for	ATGGATGAACTATACAAAGGCGCG CCGCAAACGGCAGATTTCGGCATC	N-terminal fusion of GFP
CryA N-trunc 40 GFP for	catggatgaactatacaaaGGCGCGCCgCG GTGCGAAGCTTACAACAG	N-terminal fusion of GFP
CryA N-trunc 60 GFP for	CAT GGA TGA ACT ATA CAA AGG CGC GCC GTA CAC AGA GAC GAG CGC TCA	N-terminal fusion of GFP
atfA YFPC Efi for	AAC AGA AGG TCA TGA ACC ACG GCG CGC CGA TGT CTG CCG CCG TGG CT	N-terminal fusion of YFPC
atfA YFPN Efi for	TCA TGC GCT CCA TCG CCA CGG GCG CGC CGA TGT CTG CCG CCG TGG CT	N-terminal fusion of YFPN
atfA YFP Efi rev	GTC GAC TCT AGA GGA TCC TTA ATT AAT CAA GTG TAT GGA GGA TTC GGG	N-terminal fusion of YFPC/N/GFP
CryA ∆70 NTE cutoff OE for	GTT CTC TAC TCA GTT AAT TAG GGC GCG CCA ATG ACC GCG AAC GCG GCA	Overexpression of truncated CryA
atfA GFP Efi for	CATGGATGAACTATACAAAGGCGCGCC GATGTCTGCCGCCGTGGCTT	N-terminal fusion of GFP
CryA Del Cysteine in NTE toAla for	GAA GCT TAC AAC AGC GGA AT C C	Deletion of cysteine to alanine
CryA Del Cysteine in NTE to Ala rev	ATT CCG CTG TTG TAA GCT TC G GCC CGC GCGTTG CTC ATT T CT G	Deletion of cysteine to alanine
CryA native promoter in Efimov for	aaaacgacggccagtgaattGTGCAGGATGAT CGCATCGA	Recomplementation of cryA
CryA native promoter in Efimov rev	agttcttctcctttactcatGTTCCAAGCTTGCG AAGTTTATTTG	Recomplementation of cryA
brlA_RT_Anid for	GGGACTTCCATACGGTAGCA	qRT-PCR
brlA_RT_Anid rev	TTCTGAGGGCATCCACAGTT	qRT-PCR
flbB_RT_Anid for	TGAGCGGAAGGAGCAATACA	qRT-PCR

flbB_RT_Anid rev	TTGGTGAACAGCAAGGTTCG	qRT-PCR
ccgA_RT_Anid for	CGCTTCCCTCACTTCTCGT	qRT-PCR
ccgA_RT_Anid rev	TTCTTAGCGGCCTCCTTGTG	qRT-PCR
ccgB_RT_Anid for	ATAACGCCGACCTGACTACG	qRT-PCR
ccgB_RT_Anid rev	TTGGCGGCTTCCTTGTAAAC	qRT-PCR
AN11314_RT_Anid for	GCCTACTGAGTCGCAGACAA	qRT-PCR
AN11314_RT_Anid rev	GTAGATTGTATCGGCAGGGC	qRT-PCR
conJ_RT_Anid for	ACCAGAACCCCGGTAACTTC	qRT-PCR
conJ_RT_Anid rev	CAGAGTCCATACTGGCAAAGC	qRT-PCR
H2B_RT_Anid for	GAAGAAGCGCGGAAAGACC	qRT-PCR
H2B_RT_Anid rev	TAGACATAGCACGGGTGGAG	qRT-PCR
cetJ_RT_Anid for	TTC TAA ACC CAA CTC GCC CT	qRT-PCR
cetJ_RT_Anid rev	GAT GGG CGA CTG AGA GAT GA	qRT-PCR
AN8930_RT_Anid for	TTCGACCATCCTGAGCTGAA	qRT-PCR
AN8930_RT_Anid rev	GTTTGGCTAGCTCCTCGTTG	qRT-PCR
catA_RT_Anid for	TCG AAC TGA ACC GCA ACA TC	qRT-PCR
catA_RT_Anid rev	GGG TCG TCA GAG AAG TCG AT	qRT-PCR
catB_RT_Anid for	GTGGTGTTGACTTCACCGAG	qRT-PCR
catB_RT_Anid rev	TGGGCAACTGCTCAAAGTTC	qRT-PCR

glrA_RT_Anid for	TCACGTTCACAAGAAGCACC	qRT-PCR
glrA_RT_Anid rev	ATCTCCGAGCCATCGTTCAT	qRT-PCR
trxA_RT_Anid for	GCTAAACGCGCCTTTTCATC	qRT-PCR
trxA_RT_Anid rev	TGGAATTCGGCCTTAGAGGT	qRT-PCR
prxA_RT_Anid for	GTTATGCCATCGTCCTCGAC	qRT-PCR
prxA_RT_Anid rev	GTGCTTGATGACAGTCTCGG	qRT-PCR
ccgA_ChIP_Anid for	GTGGTAATGACAGGAAAGGCC	ChIP
ccgA_ChIP_Anid rev	GAGTTCGTCATAAGCATGGGCG	ChIP
actA_ChIP_Anid for	CTTCTCAACATCCAACTCCC	ChIP
actA_ChIP_Anid rev	GGTGGATTAGAATCGAACTAC	ChIP

Supplementary References

1 Blumenstein, A., Vienken, K., Tasler, R., Purschwitz, J., Veith, D., Frankenberg-Dinkel, N. & Fischer, R. The *Aspergillus nidulans* phytochrome FphA represses sexual development in red light. *Curr. Biol.* **15**, 1833-1838 (2005).

2 Hedtke, M., Rauscher, S., Röhrig, J., Rodríguez-Romero, J., Yu, Z. & Fischer, R. Light-dependent gene activation in *Aspergillus nidulans* is strictly dependent on phytochrome and involves the interplay of phytochrome and white collar-regulated histone H 3 acetylation. *Mol. Microbiol.* **97**, 733-745 (2015).

3 Purschwitz, J., Müller, S., Kastner, C., Schöser, M., Haas, H., Espeso, E. A., Atoui, A., Calvo, A. M. & Fischer, R. Functional and physical interaction of blue-and red-light sensors in *Aspergillus nidulans*. *Curr. Biol.* **18**, 255-259 (2008).

4 Leister, K., Dong, Y., Landmark, A., Ma, Y., Schreckenberger, B., Yu, Z., Lu, L. & Fischer, R. Distinct roles of phytochromes A and B in *Aspergillus fumigatus* in environmental sensing and pathogenicity. *mBio* **16**, e02204-02225 (2025).

5 Yu, Z., Huebner, J., Herrero, S., Gourain, V. & Fischer, R. On the role of the global regulator RlcA in red-light sensing in *Aspergillus nidulans*. *Fungal Biol.* **124**, 447-457 (2020).
