# Supplementary material for: The photolyase/cryptochrome of Aspergillus nidulans senses oxidative stress and shuttles from nuclei to mitochondria
Source: Nat Commun. 2026 Feb 7;17:1483. doi: 10.1038/s41467-026-69403-2 (PMC12887071; doi:10.1038/s41467-026-69403-2)
Supplement: Supplementary file 2 — Description of Additional Supplementary Files [file 41467_2026_69403_MOESM2_ESM.pdf]

Description of Additional Supplementary Files:

Supplementary Movie 1: Shuttle of CryA to mitochondria after addition of H2O2. CryA was fused N-terminally to GFP under the control of the alcA promoter. Spores were incubated overnight at 25°C in minimal media with 2% glycerol instead of glucose to derepress the alcA promoter (moderate expression level). Hoechst staining was used for nuclei visualization. Microscopy was performed with the Axio Imager Z1 using the Objective W Plan-Apochromat 63/1.4 Oil DIC. To induce the shuttle, 1 µl of a 10 mM H2O2 solution was added directly to the sample. A total of 67 images for a total of 73 seconds were recorded. iMovie was used to convert the images into a movie.
